# Coconut (*Cocos nucifera*) Ethanolic Leaf Extract Reduces Amyloid-β (1-42) Aggregation and Paralysis Prevalence in Transgenic *Caenorhabditis elegans* Independently of Free Radical Scavenging and Acetylcholinesterase Inhibition

**DOI:** 10.3390/biomedicines5020017

**Published:** 2017-04-21

**Authors:** Rafael Vincent Manalo, Maries Ann Silvestre, Aza Lea Anne Barbosa, Paul Mark Medina

**Affiliations:** 1 Department of Biochemistry and Molecular Biology, College of Medicine, University of the Philippines Manila, Ermita, Manila 1000, Philippines; rmmanalo3@up.edu.ph; 2 Juan R. Liwag Memorial High School, Gapan, Nueva Ecija 3105, Philippines; mariesannrs@yahoo.com (M.A.S.); barbosa.azaleaanne@yahoo.com (A.L.A.B.)

**Keywords:** Alzheimer’s disease (AD), sporadic inclusion body myositis (sIBM), coconut leaf extract, *Cocos nucifera*, *Caenorhabditis elegans*

## Abstract

Virgin coconut oil (VCO) has been the subject of several studies which have aimed to alleviate Alzheimer’s disease (AD) pathology, focusing on in vitro antioxidant and acetylcholinesterase (AChE) inhibitory activities. Here, we studied an underutilized and lesser-valued part of the coconut tree, specifically the leaves, using in vitro and in vivo approaches. Coconut leaf extract (CLE) was screened for antioxidant and AChE inhibitory properties in vitro and therapeutic effects in two strains of transgenic *Caenorhabditis elegans* expressing amyloid-β_1–42_ (Aβ_1-42_) in muscle cells. CLE demonstrated free radical scavenging activity with an EC_50_ that is 79-fold less compared to ascorbic acid, and an AChE inhibitory activity that is 131-fold less compared to Rivastigmine. Surprisingly, in spite of its low antioxidant activity and AChE inhibition, CLE reduced Aβ deposits by 30.31% in CL2006 in a dose-independent manner, and reduced the percentage of paralyzed nematodes at the lowest concentration of CLE (159.38 μg/mL), compared to dH_2_O/vehicle (control). Phytochemical analysis detected glycosides, anthocyanins, and hydrolyzable tannins in CLE, some of which are known to be anti-amyloidogenic. Taken together, these findings suggest that CLE metabolites alternatively decrease AB_1–42_ aggregation and paralysis prevalence independently of free radical scavenging and AChE inhibition, and this warrants further investigation on the bioactive compounds of CLE.

## 1. Introduction

Alzheimer’s disease (AD) afflicts more than 26 million people globally, and is said to be the most common neurodegenerative disease worldwide. It is mainly idiopathic, with late-onset affecting more than 90% of cases [1]. It is classified, across its pathological hallmarks, as a tauopathy-characterized by hyperphosphorylated, filamentous tau aggregates prior to microtubule collapse—a major requisite for the formation of neurofibrillary tangles [2,3]. The presence of amyloid-β (Aβ) plaques are thought to constitute the main biomarkers for AD. The combined criteria from CERAD, Braak NFT, and Thal, for instance, maintain the definition of AD as a procession from a complex of clinically pathological diagnoses of Aβ aggregates, neurofibrillary tangles, and cognitive dysfunction; however, recent case series showing AD-diagnosed patients lacking Aβ deposits challenge the generalizability of the criteria, as well as the role of protein aggregates in the development and progression of AD [4]. Nonetheless, the combined presence of tangles and plaques, which are associated with progressive dementia and neurodegeneration, is the most widely accepted view [5].

Interestingly, the presence of Aβ and tau pathologies in AD are said to associate with inflammatory responses, the latter paving the way to the development of the disease in question. The soluble tau oligomers, which are hypothesized to bring about more drastic adverse events related to tangle formation, were shown to co-localize with inflammation-associated astrocytes, microglia, and related cytokines [3]. In addition, a recent study by Laurent et al. showed that T-cells from the hippocampus mediate the inflammation process and promote cognitive decline [6]. What is alarming is the fact that tau proteins propagate their pathological assemblies in a prion-like manner [7], which tends to aggravate the degree of inflammation and progression of AD.

Remarkably, the symptoms and general features of AD coincide with that of sporadic inclusion body myositis (sIBM)—a form of skeletal muscle disease. As with AD, sIBM is by consensus a combination of muscular degeneration and inflammation [8], often characterized by slow-onset atrophy, lethargy, and dysphagia, with hallmark biomarkers of filamentous inclusions and intracellular Aβ deposits in muscle cells [9]. As with AD, sIBM is late-onset, with the highest prevalence occurring in older age groups, especially beginning at the age of 50 [8,10]. Thus, it is seen that both degenerative diseases contribute to lessening the quality of life of the elderly, aggravated by the lack of a known cure.

There have been many researches on VCO and its potential to salvage neurons from amyloid-induced degeneration, reduce inflammation, and provide ketone bodies for therapeutic effects and increased cognitive function [11,12,13,14]. However, it is proposed that the anti-amyloidogenic and anti-aggregatory properties are to be found in the phenolic compounds of the plant [15,16], which motivated the present study. Here, we demonstrate the antioxidant and acetylcholinesterase (AChE) inhibitory properties of coconut leaf extract (CLE) using 2,2-diphenyl-1-picryl-hydrazyl (DPPH) scavenging and AChE inhibition assays. We then demonstrated that CLE reduced Aβ aggregation and paralysis in vivo. Phytochemical analysis then revealed that glycosides, anthocyanins, and hydrolyzable tannins were present in the extract, warranting further investigation on these bioactive compounds in CLE.

## 2. Materials and Methods

### 2.1. Leaf Harvesting and Crude Ethanolic Extraction of Cocos nucifera Leaves

Leaves were harvested from mature coconut (*Cocos nucifera)* trees in ecologically acceptable proportions at San Nicolas, Gapas City of the Nueva Ecija province, Philippines. For confirmation and the correct classification of plant identity, samples were sent to the botany division of The National Museum at Ermita, Manila, Philippines, and to the Industrial Technology Development Institute (ITDI) of the Department of Science and Technology (Taguig, Philippines) for standardized crude ethanolic extraction. For phytochemical analysis, the same outsource implemented the procedure, as requested.

### 2.2. Caenorhabditis elegans Strains

All strains were obtained from and provided by the Caenorhabditis Genetics Center (CGC) of the University of Minnesota, which is funded by the NIH Office of Research Infrastructure Programs (P40 OD010440). For the present study, the following strains were used:

N2—wild type
C. elegans var. Bristol
CL4176—expresses Aβ_1–42_
dvIs27 [myo-3p::A-β(1-42)::let-851 3′UTR]
CL2006—expresses Aβ_1–42_; temperature-sensitive
dvIs2 [pCL12(unc-54/human Aβ peptide 1-42 minigene) + pRF4]

CL2006 strains were maintained at 20 °C, while CL4176 strains were kept at 16 °C, and both were periodically transferred to OP50-incubated nematode growth media (NGM) plates. For phenotypic activation in CL4176, heat-sensitive nematodes were continually exposed at 25 °C post-treatment. All plates were prepared according to the protocol of Steirnagle [17].

### 2.3. Antioxidant and Acetylcholinesterase Inhibitory Activities of Crude CLE Extracts

Leaf extracts were tested for their antioxidant properties and capability of inhibiting AChE via the DPPH and AChE inhibition assays, respectively. For the DPPH scavenging assay, five treatments of CLE with progressing concentrations (in μg/mL) and one control were prepared, as follows. For the positive control, ascorbic acid at the same concentrations (in μg/mL) as the CLEs were tested. On the other hand, methanol was used as the negative control. Absorbance was then taken after 30 min at 517 nm and dose-response curves for both CLE and ascorbic acid were graphed, from which the EC_50_ value was directly obtained. The EC_50_ is the effective concentration where a half-maximal effect is observed. The AChE inhibition assay was similarly performed, consisting of 11 treatment groups. The positive control used in this assay was Rivastigmine, which is a known AChE inhibitory drug and is currently used as a treatment for patients with AD [18]. Meanwhile, the protocol for the AChE inhibitory assay was adopted from Ellman et al. [19]. Enzyme activity was obtained at 25-second intervals for 31 readings at 420 nm, using the Thermoscientific Multiskan. Both assays were done in triplicate.

### 2.4. Nematode Toxicity Assay

To eliminate the confounding factor of toxicity in the procedure, nematodes – wild-type (WT) and transgenic (TG) – were exposed to increasing concentrations of CLE (12.75, 127.5, 1275 μg/mL). Further, nematodes were exposed to sterile dH_2_O as the negative control. All treatment groups were exposed to intervention for 24 h, adopted from Qiao et al. [20], after which the percent mortality was obtained using a stereoscope from the Department of Biochemistry and Molecular Biology, College of Medicine, University of the Philippines Manila (Manila, Philippines). The toxicity assay aforementioned was done in triplicate.

### 2.5. Amyloid-β Aggregation and Paralysis Tests

To test the protective effect of CLE against AD and sIBM in vivo, two *C. elegans* strains (CL2006 and CL4176) were exposed to varying concentrations of CLE. In all procedures, sterile dH_2_O acted as the negative control. For CL2006, nematodes were divided into groups and exposed to treatment at 20 °C for five days. Nematodes were then sampled via worm-picking and placed on 2% agarose pads with two drops of ~80% glycerol, before being placed on a microscope slide. At this point, some of the worms may perish, so the glycerol may be lowered to ~50% or be viewed within five minutes or less to prevent confounding degradation. Aβ deposits were viewed under bright-field microscopy to improve protein deposit scoring. For CL4176, treatment was administered prior to heat activation. Nematodes were divided into groups and exposed at different concentrations of CLE at 16 °C for 36 h. The temperature was then raised to 25 °C for two hours, and the proportion of paralyzed nematodes was obtained every 12 for 36 h. Likewise, the aggregation assay was employed using similar conditions to the paralysis assay (Table 1). For both strains, the assays were done in triplicate.

### 2.6. Statistical Analyses

The effects of intervention and control were compared via analysis of variance (ANOVA) and IC_50_ and EC_50_ were determined using GraphPad Prism 6.0 (GraphPad Software, Inc., La Jolla, CA, USA). For all of the statistical tests, a *p*-value of *p* < 0.05 was accepted as statistically significant. To further test the validity of the results, post-hoc two-tailed t-tests (in least significant difference) were computed where applicable.

## 3. Results

### 3.1. Coconut Leaf Extract (CLE) Neutralizes DPPH Radicals in a Dose-Dependent Manner

**Figure 1:**
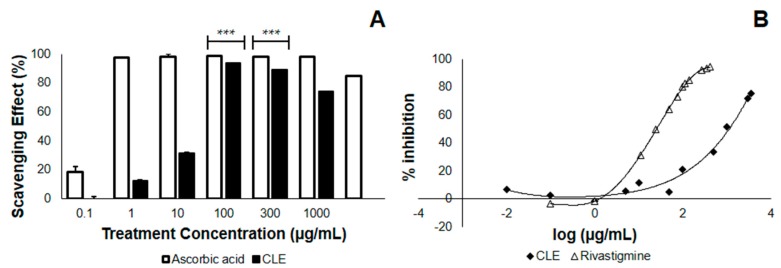
2,2-diphenyl-1-picryl-hydrazyl (DPPH) scavenging and acetylcholinesterase (AChE) inhibition by ethanolic coconut (*Cocos nucifera)* leaf extract in vitro. The CLE was tested at varying concentrations in vitro for antioxidant activity and the ability to inhibit standard AChE. (**A**) DPPH scavenging effect of CLE compared with the control (ascorbic acid). At 100 μg/mL, CLE neutralized DPPH radicals by 93.85% (*n* = 3). *** *p* < 0.0001 when compared with the control at the same concentration using a two-tailed *t*-test. *p* < 0.05 was used to compare treatment groups 4 to 6 (100–1000 μg/mL) using one-way ANOVA; (**B**) Dose-response curves comparing the AChE inhibitory activity of CLE and positive control (Rivastigmine) in vitro.

In the study conducted, leaves from *Cocos nucifera* were first tested in terms of their antioxidant property and AChE inhibitory activity, before being tested in vivo, for practical purposes. Here, we checked whether the CLE would neutralize the DPPH radical, thus lowering its absorbance at 517 nm. We found that at the λ_max_ of DPPH, CLE neutralizes DPPH with an EC_50_ of 18.11 μg/mL (Table 2), less than one-fifth of the concentration of the 4th treatment group (Figure 1A). This implies that at a relatively low concentration, CLE elicits an antioxidant effect. Further, we found that the antioxidant property of CLE in vitro was dose-dependent, in that higher concentrations of CLE led to greater losses of DPPH absorbance at 517 nm (Figure 1A). Apparently, there was no difference in the antioxidant activity between 100–1000 μg/mL, as confirmed by one-way analysis of variance (ANOVA). However, the antioxidant activity at these concentrations might have already been saturated. In terms of its EC_50_, the antioxidant activity of CLE was about 79-fold less—implying a possible contribution from antioxidant activity against Aβ that is less manifold than if it were ascorbic acid.

### 3.2. CLE Inhibits AChE Less Effectively than Does Rivastigmine In Vitro

We then checked whether CLE inhibits AChE, which is postulated to confer protection from complications in AD and is therefore a desired effect. The results showed that when CLE is homogenized with AChE and incubated at 25 °C for 15 min, it inhibits the activity on acetylcholine metabolism with an IC_50_ of 3218.56 μg/mL. Using the standard of treatment (Rivastigmine), the IC_50_ value was found to be 24.52 μg/mL (Figure 1B). This implies the presence of an inhibitory activity only at manifold higher concentrations, as compared with vehicle (Table 2).

### 3.3. CLE is Non-Lethal to Wild-type and Transgenic C. elegans CL2006 and CL4176

To eliminate the possibility that CLE has intrinsically deleterious effects in vivo, we exposed strains N2, CL2006, and CL4176 at concentrations progressing to three orders of magnitude (12.75, 127.5, 1275 μg/mL). Observations post-treatment showed a 100% survival rate for all nematodes, indicating that at these concentrations, any lethal effects that are observed are solely due to Aβ_1–42_ expression.

### 3.4. CLE Significantly Reduces Aβ Aggregate Deposits in Transgenic C. elegans Strains CL2006 and CL4176

We next determined whether CLE would elicit protective effects in vivo. Varying concentrations of CLE were administered to groups of transgenic *C. elegans* after a maturation period of three days; then, measurements were successively taken post-treatment, according to established protocol. For testing the therapeutic effects of CLE, a *C. elegans* strain population continuously expressing Aβ aggregates in the muscle cell walls (CL2006) was used.

**Figure 2:**
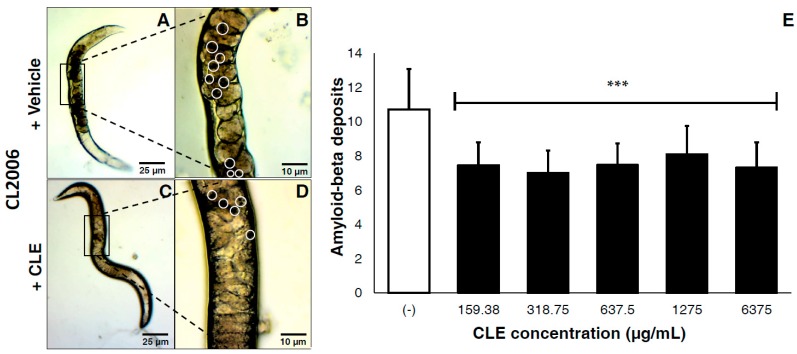
CLE significantly reduces amyloid-β plaque deposits in transgenic *C. elegans* strains CL2006, independently of concentration. Nematodes were transferred to OP50-incubated NGM plates and allowed to mature for three days at 20 °C, after which they were fed with either vehicle or CLE at varying concentrations *ad libitum*. The amyloid-β deposits in CL2006 were then counted five days post-treatment, and were pooled in triplicate. Dark portions in the cell wall of *C. elegans* indicate the protein deposits. CL2006 strains were fed with either (**A**) vehicle or (**C**) CLE. Detailed images of the body walls of nematodes fed with (**B**) vehicle or (**D**) CLE are shown. White circles identify the aggregate deposits; (**E**) Aβ deposits were counted per treatment group at varying concentrations of CLE (in μg/mL). *** *p* < 0.0001 when compared with vehicle using two-tailed *t*-test. *p* < 0.05 was used to test for significance between treatment groups using one-way ANOVA.

This tested the efficacy of CLEs against plaque deposition after the formation of Aβ proteins. In vivo, CLE protected against Aβ toxicity by reducing the deposits of visible aggregates in the cell walls of CL2006 (Figure 2A–D). Compared to vehicle, a mean reduction of 30.31% in Aβ deposits was observed. Two-tailed *t*-tests confirmed this observation as highly statistically significant at *p* < 0.0001 (Figure 2E). Further, there were no significant differences observed on the deposition of Aβ aggregates, regardless of the CLE concentration administered. This implies that the efficacy of CLE is somewhat dose-independent.

### 3.5. Paralysis of CL4176 C. elegans Strains Was Partially Relieved by Exposure to CLE Prior to Heat Activation of Aβ Expression

**Figure 3:**
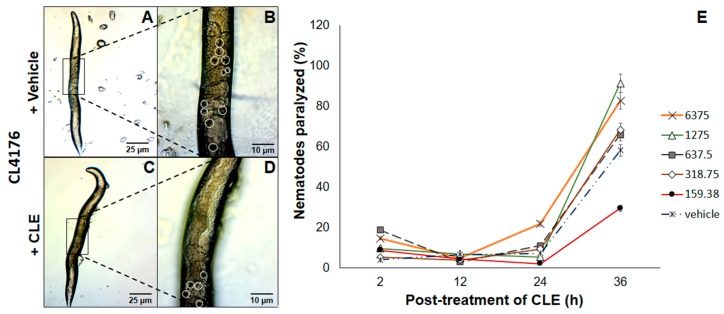
CLE reduces paralysis prevalence in *C. elegans* strain CL4176. Nematodes were exposed to (**A**) vehicle or (**C**) CLE in concentrations similar to the Aβ aggregation assay in vivo. Aβ expression was induced at 25 °C after 36 h post-treatment of CLE or vehicle at 16 °C for 36 h. Detailed images of the body walls of nematodes fed with (**B**) vehicle or (**D**) CLE are shown. White circles identify the aggregate deposits; (**E**) The proportion of paralyzed nematodes were gathered at 2, 12, 24, and 36 h post-induction. To determine the link of aggregation in paralysis, the aggregation assay was done in CL4176 using the paralysis assay conditions.

The next question to ask was whether CLEs elicited beneficial effects when administered at an earlier time point. Therefore, a strain only expressing Aβ proteins upon exposure to a temperature of 25 °C and above proved the utility. To this end, CL4176 *C. elegans* strains were exposed to CLE for 36 h, before allowing Aβ production. This is in stark contrast with CL2006, primarily because the prophylactic effect of CLE was now being tested. In all of triplicates examined, nematodes assigned to treatment groups with CLE exposure had a lower prevalence of paralysis at the 12th hour post-induction, which then led to paralysis no better than or worse than the control, except at the concentration of 159.38 μg/mL (Figure 3E).

To assess the link between aggregation and paralysis, the CL4176 strain was used for the aggregation assay at the concentration with the lowest apparent effect in CL4176 (1275 μg/mL) at the 36th hour, with conditions similar to the paralysis assay. As the results showed, aggregates were lessened by up to 54.55% compared to vehicle at 12 h post-induction. To this end, it is likely that paralysis reduction was drastic at this time point due to a high reduction in Aβ deposits—however, the lack of a trend between paralysis and Aβ aggregation at the 24th and 36th hours suggests that CLE can only delay paralysis, and that Aβ aggregation is not the only determinant of paralysis in CL4176, since the concentration that worked was the lowest (159.38 μg/mL), which should also have the lowest antioxidant and AChE inhibitory activities of all the CLE treatment groups. The observed proportion of nematodes relieved of motor deficits was statistically confirmed with a value of *p* < 0.05.

There was a certain difficulty in distinguishing the proportion of relieved nematodes using the CL2006 strains, so the paralysis assay was only done on CL4176. This difficulty is in fact warranted, due to the inherent resistance of CL2006 to paralysis—indeed, the variability of paralysis between nematodes was found to be unpredictable, with some nematodes never becoming paralyzed [21]. Hence, the need to use CL4176 for paralysis assays related to Aβ expression maintains its essentiality.

### 3.6. Phytochemicals Present in CLE Possibly Interact with Aβ_1–42_ Peptide

In the phytochemical analysis, screening of the compounds resulted in the presence of glycoside compounds, flavonoids (particularly anthocyanins), and hydrolyzable tannins (Table 3). Due to the presence of hydrolyzable tannins in the CLE, it was postulated that the metabolites of such tannins could also contribute to the efficacy of the leaf extract. Urolithin A, a derivative of ellagitannin that is known to induce mitophagy and increase the lifespan of *C. elegans* [22,23,24,25]; gallic acid, a part of hydrolyzable tannins with known antimicrobial, antioxidant, and neuroprotective effects [26,27,28]; and pyrogallol, a derivative of gallic acid whose moiety is associated with β-secretase (BACE1) inhibition [29] and together with gallic acid is known to be anti-amyloidogenic [30], could be possible contributors to the protective effect of CLE in vivo. It was noted that cardiac glycosides may accumulate in the central nervous system [31], warranting investigation on its alternative yet promising effect on Aβ_1–42_ aggregation.

## 4. Discussion

### 4.1. C. elegans as a Model for Alzheimer’s Disease (AD) and Sporadic Inclusion Body Myositis (sIBM)

In the study conducted, Aβ aggregation and its toxicity in the form of paralysis were treated in vivo with ethanolic extracts from coconut (*Cocos nucifera*) leaves. The transgenic *Caenorhabditis elegans* strains used served as biological models of AD and sIBM—the former in terms of Aβ overexpression and aggregation, and the latter in terms of expression in the muscle cell walls. Further, the two strains modelled the effects of administration at two different time points-before the induction of Aβ expression and after it was expressed. While higher forms of animal models would generate greater external validity, the use of transgenic nematodes for this study holds several advantages. For one, human orthologs of disease genes such as tau, relevant neuronal cells, ion channels, and transporters are conserved in *C. elegans*, which are important in neurodegenerative disease studies.

To this end, nematodes have been utilized in pathway analysis and drug screening for diseases such as AD, Parkinson’s disease (PD), and Huntington’s (HD) [32,33,34]. Further, the conservation of 12 over 17 signalling cascades, a short generation time, and a life cycle of two to three weeks, as well as the ease of observation provided by its transparent body lining, provide efficient tools for scoring protein aggregation [35]. While it is arguable that aggregation in muscle walls does not entirely represent the multiple factors associated with AD, expression in muscle cells grants a larger picture of protein formation, and is useful for studies specifically targeting insoluble Aβ deposits, since it is easier to visualize than if it were in neuronal cells and is actually the tissue of choice for studies involving such proteins [36,37].

### 4.2. Reduction of Aβ_1–42_ Aggregation by CLE Is Independent of Free Radical Scavenging and AChE Inhibition

In the DPPH assay, the EC_50_ value for radical scavenging was found to be 18.11 μg/mL, and as the concentration increased further, the antioxidant activity remained the same, as confirmed by ANOVA. However, this antioxidant activity may or may not play a part in the amelioration of insoluble Aβ deposits, due to the fact that CLE has a ~79-fold greater EC_50_ as compared to ascorbic acid (Table 2).

In the AChE inhibition assay, CLE showed even less effective results. In preventing the activity of standard AChE, the IC_50_ of CLE was found to be more manifold than that of Rivastigmine (3218.56 μg/mL versus 24.52 μg/mL), implying a dose-dependent activity whose efficacy is observed at much higher concentrations. The critical results were manifested in the Aβ aggregation assay in CL2006. In all treatment groups (159.38.5 to 6375 μg/mL), a steady decrease in protein deposition of 30.31% was observed. Strikingly, this opposed the dose-dependent activity of CLE against AChE, since at every concentration from 160 μg/mL, the efficacy of the extract was consistently maintained. Were AChE inhibition to contribute significantly, the beneficial effect should be observed between treatments 4 and 5 (1275 and 6375 μg/mL)—however, this was not the case. Hence, CLE acted against Aβ deposits through a pathway that involved more than just AChE inhibition.

Further, the results opposed, in some way, the antioxidant activity of CLE against DPPH. CL2006 displayed a 30.31% Aβ reduction from 187.5 μg/mL of CLE, which was also dose-independent and was statistically the same throughout all the concentrations. While this concentration is about nine times that of the EC_50_ value in vitro, we argue that: (1) the composition of CLE as it acts in vivo would likely change due to metabolism by the *C. elegans* machinery; and (2) it is expected that at high concentrations, vitamin C, as well as other antioxidants, should become pro-oxidant in vitro and in vivo [22,23,24]—therefore, at least at treatments 3 to 5 (with concentrations 35 to 350 times higher than EC_50_ in vitro), Aβ was expected to increase. It is worth noting that previous in vivo studies demonstrated the therapeutic effects of vitamin C at moderate doses [38,39], which at larger doses of about 600 mg/kg, resulted in increased neurodegeneration and neuroinflammation in AD rat models [40]. These findings reflect the duality of vitamin C in terms of its oxidizing ability [41,42], which may promote Aβ deposition by reacting with metal ions similar to how copper (Cu^2+^) induces Aβ-mediated H_2_O_2_ generation upon binding to His residues and undergoing redox reactions [43]. Since CLE is non-inferior to ascorbic acid in terms of its antioxidant activity at high concentrations, a similar trend to previous studies should be reflected in the treatment groups, which were 9–350 times higher than the DPPH EC_50_ of CLE. Strikingly, this was not the case, and Aβ deposition neither increased nor decreased significantly at higher CLE concentrations in vivo. To this end, we hypothesize that CLE is also acting in a way that opposes the free radical scavenging duality at high concentrations.

**Figure 4:**
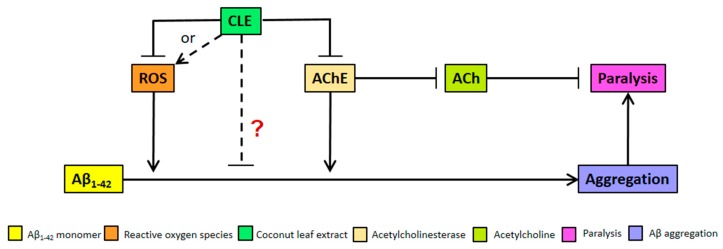
Model for Aβ aggregation and paralysis reduction by CLE in transgenic *C. elegans*. CLE acts by inhibiting reactive oxygen species (ROS) and AChE activities in *C. elegans*, albeit at high concentrations—both of which would otherwise worsen Aβ-induced pathology. However, at high concentrations, a pro-oxidant effect is expected due to antioxidant excess, resulting to CLE-induced ROS production (broken arrow). At concentrations 9 to 350 times higher than the EC_50_ of CLE, the effect on aggregation was dose-independent, as shown in Figure 2. Therefore, it is possible that CLE is acting directly through compounds that are anti-aggregatory, which masks the pro-oxidant activity of antioxidants in CLE at higher extract concentrations (red “?”).

We clarify that the alternative anti-aggregatory effect of CLE might be dose-dependent, but is being masked by the increasing pro-oxidant activity of antioxidants at higher concentrations of CLE (Figure 4). This hypothesis is not unlikely, since recent studies have also shown neuroprotective effects that are either independent of antioxidant activity or address a more complex interplay of factors that constitute an antioxidant, such as lipophilicity and molecular weight [44,45]. These remarkable results point out the possible mode of action of CLE metabolites apart from conventional criteria, such as interactions with Aβ-related proteins like the peroxisome proliferator-activated receptor gamma (PPARγ) and pro-apoptotic proteins—respectively known to be upregulated and downregulated by natural products such as epigallocatechin gallate (EGCG) in green tea [29]. Further, CLE may instead be interacting with known proteins affecting Aβ deposition in *C. elegans*, such as DAF-2 and FOXO [46,47], all of which warrant further investigation on the possible mechanisms on CLE metabolites.

We hypothesize, therefore, that bioactive compounds in CLE directly act to inhibit Aβ_1–42_ aggregation (Figure 4), and if these anti-aggregatory compounds in associated CLE are isolated and separated from those that confer protection from oxidative stress, a much greater effect might be observed in vivo.

### 4.3. Reduction of Aβ-Induced Paralysis by CLE Is Independent of Free Radical Scavenging and AChE Inhibition

In the paralysis assay, CL4176 nematodes displayed a higher incidence of paralysis 2 h post-induction in treatment groups with CLE. The hypothesis that CLE is deleterious in terms of paralysis was tested by comparing the proportion of nematodes paralyzed 12 h post-induction. The results showed that this was not the case. In the assays performed, CL4176 nematodes were relieved of paralysis compared to the control at the lowest concentration of CLE (159.38 μg/mL). Since this concentration also exhibits the lowest antioxidant and AChE inhibitory activities, the effect observed against paralysis implies an action independent of antioxidant activity and AChE inhibition. From here, it may be inferred that the factors determining the incidence of paralysis are different from those that aggravate its progression. For instance, it is known that the lifespan of *C. elegans* is short (two to three weeks), more so with transgenic strains, and that as the nematodes age, the capacity to maintain proteostasis decreases, aggravating the accumulation of insoluble proteins that exist even in physiologic conditions [48,49]. Therefore, it is rational to hypothesize that the extracts might also be increasing the lifespan of the paralyzed nematodes. Should this be the case, then the succeeding cross-sectional observations would tend to obtain a higher proportion of paralyzed nematodes in the CLE treatment groups, because more nematodes survive long enough to be counted and these nematodes, due to an older age, develop a more profound form of paralysis. These results may have implications in the age and concentration at which CLE administration would elicit its maximum protective effect against AD and sIBM. Further, it is also possible that CLE has bioactive compounds which are cytotoxic but non-lethal to the nematodes at very high concentrations, but was not readily evident in the aggregation and nematode toxicity assays because of the suspected anti-aggregatory bioactive compound.

We further compared the effects of CLE exposure in CL2006 and CL4176, and found that the concentration of CLE does not matter in CL2006; rather, at any concentration, the nematodes benefited in terms of the reduction in Aβ deposits. With CL4176, this was not the case. This is implicative of two possibilities: (1) CLE contains a bioactive compound that is exclusively effective against Aβ_1–42_ aggregation; and (2) more factors other than Aβ aggregation, ROS production, and AChE action on ACh may exist in the progression of paralysis in *C. elegans*. We recommend using a greater sample size than *n* = 30 to assess whether the paralysis trend improves, as well as using higher animal models, such as mice.

### 4.4. Anthocyanins, Tannins and Glycosides Are Candidate Compounds Against Aβ_1–42_ Induced Pathology

Phytochemical screening of CLE showed the presence of anthocyanins, hydrolyzable tannins, and glycosides. It is important to note that the metabolism of CLE by *C. elegans* occurred throughout the study; hence, the isolation of a single compound might be more complex. For instance, secondary metabolites such as urolithin A, which has been shown to extend the lifespan of *C. elegans* and improve muscle strength [22,23,24,25], or gallic acid and its derivative pyrogallol that are known as anti-amyloidogenic compounds [30], are all known secondary metabolites of the phytochemicals detected and may in part explain the observed differences in the assays performed. Indeed, the metabolism of phytochemicals in vivo may play a role in the differential effects of CLE in vitro and in vivo.

## 5. Conclusions

AD and sIBM are neurodegenerative and inflammatory muscle diseases, respectively, but are related to each other due to known risk factors related to proteinopathies. One such factor is the aggregation of Aβ, which allows a “two birds with one stone” approach to drug or extract screening for cell-distinct diseases. The results of our study show that CLE has an antioxidant activity that is inferior by 79-fold to ascorbic acid, and an AChE inhibitory activity 131-fold less than Rivastigmine, a known AChE inhibitory drug being prescribed to AD patients. However, stark contrasts between the antioxidant and AChE inhibitory activities in vitro, and the protective effect of CLE against Aβ aggregation and paralysis in vivo, suggest that CLE may act against AD and sIBM in a way that is independent of free radical scavenging and acetylcholinesterase inhibition. However, the protective effects of CLE only delay the progression of paralysis and cannot fully salvage the nematodes from deleterious motor deficits. This warrants further investigations on the time-dependence of CLE administration and biofunctional activities apart from AChE inhibition and free radical scavenging. Lastly, the presence of anthocyanins, hydrolyzable tannins, and glycosides direct future researchers to a guided screening of compounds related to these in an effort to treat Aβ_1–42_-induced pathology.

## Figures and Tables

**Table 1:** Coconut leaf extract (CLE) treatment groups for Aβ_1–42_ aggregation and paralysis assays.

Treatment Group	CLE Concentration (μg/mL)	*C. elegans* Strain	Temperature
Aβ_1–42_ Aggregation Assay			
1	(−)	CL2006	20 °C
2	159.38		
3	318.75		
4	637.5		
5	1275		
6	6375		
Aβ_1–42_ Paralysis Assay			
1	(−)	CL4176	Pre-treatment period: 10–16 °C Post-treatment period: 24 °C
2	159.38		
3	318.75		
4	637.5		
5	1275		
6	6375		

**Table 2:** Scavenged DPPH free radicals and inhibited AChE at various concentrations of CLE.

Treatment Group	Concentration (μg/mL)	EC_50_/IC_50_ (μg/mL)		Positive Control
DPPH Scavenging Assay				
		CLE	Control	Ascorbic acid
1	0.1	18.11	0.23	
2	1			
3	10			
4	100			
5	300			
6	1000			
AChE Inhibition Assay				
1	0.01	3218.56	24.52	Rivastigmine
2	0.1			
3	1			
4	5			
5	10			
6	50			
7	100			
8	500			
9	1000			
10	3000			
11	3500			

EC_50_/IC_50_: the concentration at which a half-maximal response is observed, and depends on context.

**Table 3:** Phytochemical analysis of crude ethanolic CLE.

Test Parameter	Method	Result
Alkaloids	Mayer/Meyer test	Alkaloids absent
Anthraquinones	Bornträger test	Anthraquinones absent
Cardenolides and bufadienolides	Keller-Kiliani test	Glycoside compounds present
Flavonoids	Bate-Smith and Metcalf test	Anthocyanins present
Tannins and polyphenolic compounds	Ferric chloride test	Hydrolyzable tannins present
Saponins	Froth test	Saponins absent
